# Impact of Online Courses on University Student Visual Attention During the COVID-19 Pandemic

**DOI:** 10.3389/fpsyt.2022.848844

**Published:** 2022-03-29

**Authors:** Qi Gao, Sining Li

**Affiliations:** ^1^School of Economics, Nankai University, Tianjin, China; ^2^School of Marxism, Nankai University, Tianjin, China; ^3^College of Artificial Intelligence, Nankai University, Tianjin, China

**Keywords:** COVID-19, attention bias, eye movement, university student, cognitive theory

## Abstract

**Background:**

Under the threat of COVID-19, many universities offer online courses to avoid student gatherings, which prevent teachers from collecting responses and optimizing courses. This work collected eye movement data to analyze attention allocation and proposed instruction for improving the courses.

**Methods:**

Subjects were recruited to watch three online courses. Meanwhile, their eye movement data were collected through Dikablis Glasses. Mayer’s multimedia cognitive theory was adopted to discriminate the pivotal components of online course, and the Mann–Whitney relevance analysis demonstrated that different representations of courses affected the viewers’ attention differently.

**Results:**

Three subjects watched three different types of political courses. Course 1, which combined text and explanation, attracted the most attention. Course 2 was shown to be less attractive than course 1 and better than course 3, but the subjects were distracted by the animations in course 2. Course 3, which did not use any technique to present learning content, attracts the least attention from the subjects. A correlation analysis shows that course 1 and course 3 have similar results compared with course 2.

**Conclusion:**

Online courses have become a norm during the COVID-19 pandemic. Improving the quality of online courses can effectively reduce the impact of the epidemic on teaching. These experiment results suggest that text + commentary in the design of online courses can effectively attract the attention of the listeners and achieve better learning results. Attention gradually rises in the early stage and then falls after reaching a peak. At this time, the proper introduction of animation can effectively reverse the attention curve, while individual text or commentary results in quickly losing the listener’s attention.

## Introduction

After the sudden outbreak of the COVID-19 epidemic in early 2020, almost all stages of educational institutions decided to support online courses for students in response to the shutdown of campuses (1–3). Over 22.59 million university students and 1.67 million teachers participated in online courses (4). However, there are flaws in online learning,

such as delay in responses, lack of a sense of community and feelings of isolation, and undermined teaching quality (5–7). Traditional methods, which were used to estimate the quality and efficiency of offline courses, encountered limitations in online courses (8–10). It is also difficult for teachers to receive responses from students directly and optimize their course structures (11–14). Brown and Krzic (15) compared three different methods of teaching and taught using videos, laboratory manuals, and assignments, and combining synchronous and asynchronous and virtual laboratories were fundamental and the critical first step in transitioning to online teaching and learning. Tang et al. (16) used Fuzzy Comprehensive Evaluation-Analytic Hierarchy Process (FCE-AHP) to quantitatively evaluate the quality of online courses and divided the evaluation criteria into four major indicators and 14 s-level indicators. Hou et al. (17) created a way to investigate students’ perception of online courses in terms of teaching presence, cognitive presence, and online modality. Chick et al. (18) proposed several innovative solutions, including the flipped classroom model, online practice questions, and teleconferencing in place of in-person lecture to bridge the educational gap for surgical residents during this unprecedented circumstance. Zhang and Liu (19) investigated the college students’ attitude toward online courses in shaping their psychological distress during the COVID-19 epidemic. Yu-Fong Chang et al. (20) provided a questionnaire-based online survey to analyze the difference in learning effectiveness between a physical classroom and online class learning for dental students. Zhang and Wei (21) analyzed the positive and negative effects of online teaching mode on the effectiveness of moral education function of ideological and political courses in colleges and universities by literature research, questionnaire survey, and interview. They suggested that cultivating teachers, improving the information technology level, and expanding the Internet coverage were necessary methods. Bao (22) proposed five principles of high-impact teaching practice to effectively deliver large-scale online education.

The cognitive load theory and the cognitive theory of multimedia learning were proposed as instruction for many researchers in educational video design (23–25). Well-designed online courses influenced and promoted the students’ depth of learning (26). Iorio-Morin et al. (27) identified four workflows to improve the effectiveness of using a video in medical education based on the cognitive theory of multimedia learning. Liu et al. (28) proposed four modes of video to test whether this theory applied in English-as-a-second-language (ESL) students. Luzón and Letón’s results suggested that appropriate animation promoted selecting information, building representation models, and making sense (29).

## Materials and Methods

### Experiment Design

**FIGURE 1:**
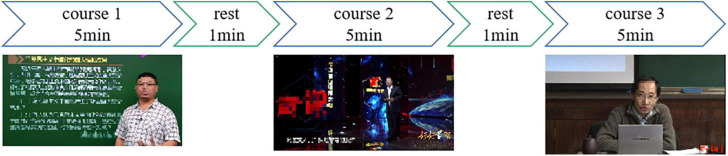
Schematic of the visual attention experiment.

Three subjects were recruited to participate in this experiment. They were required to watch three political videos which lasted 5 min each, and they had 1 min to rest between two courses. The schematic of the experiment is shown in Figure 1. The online courses can be divided into three types. Course 1 had set the text to the left of screen, and teacher stood to the right. Course 2 used subtitles to replace text at the bottom of the screen and added some transition animations to switch shots. In course 3, the teacher sat in the middle of the screen without any subtitle or animation.

### Eye Movement Tracking System

**FIGURE 2:**
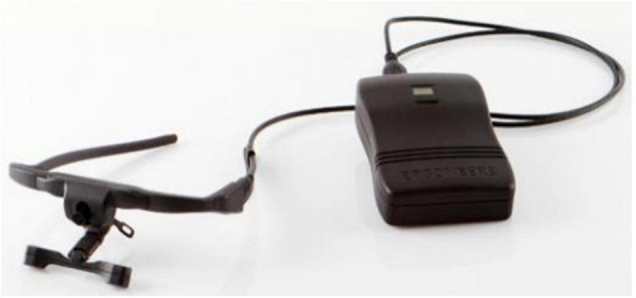
Dikablis Glasses.

**FIGURE 3:**
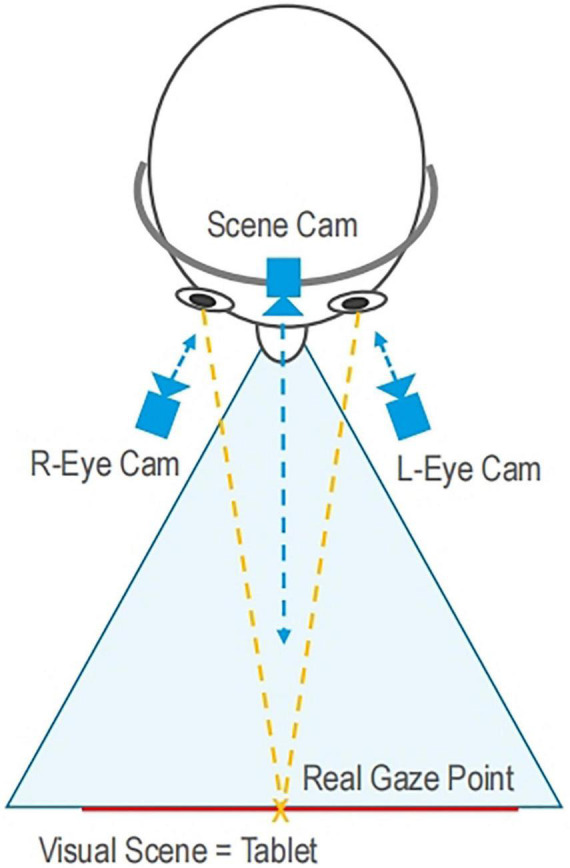
Schematic of eye movement tracking.

Dikablis Glasses system (Ergoneer, Germany) was used to collect eye movement data, which is shown in Figure 2. The full image measurement frequency of the system is 50 Hz. The horizontal viewing angle ranges from 50° to 115°, and the vertical viewing angle ranges from 50° to 115°. As shown in Figure 3, scene camera received the images of the subjects’ visual scene. The right and left eye cameras caught the position of the pupils and calculated real gaze position.

**FIGURE 4:**
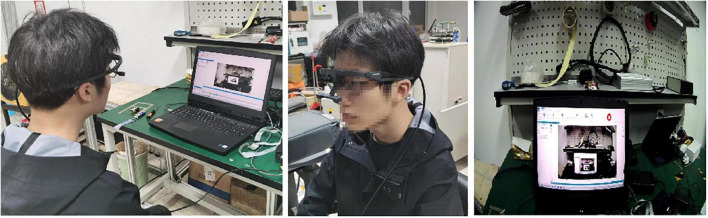
Collection of the subject’s eye movement data.

All subjects sat in front of the computer and wear eye trackers. The eye track system needed to be calibrated firstly. The gaze point was indicated by a red dot on the screen during calibration. The subject watched the online courses after adapting to the track glass, and the tracker system tracked and recorded the change of the subject’s gaze point in the field of vision in real time. The experiment process is shown in Figure 4.

### Mayer’s Multimedia Cognitive Theory

**FIGURE 5:**
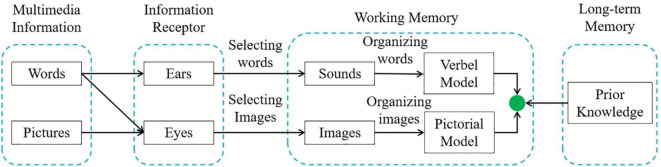
Schematic of multimedia cognitive theory.

Mayer’s multimedia learning theory is shown in Figure 5, which distinguishes multimedia information from visual information and auditory information from the perspective of sensory channels. People’s processing of the presented information relies on two channels: one is the visual channel, and the other is the auditory channel. Multimedia materials are divided into text and pictures. The information of text materials is received by the ears and the eyes, and the picture information is received by the eyes. The two channels respectively identify and classify information, accept the appropriate type of information for processing, and integrate learning with prior knowledge in long-term memory.

### Correlation Analysis Method

Mann–Whitney’s *U*-test is a non-parametric procedure in comparing two independent sample distributions (30). This method is more robust than *T*-test and can be applied to continuous data. It is especially useful when the normality assumption is not met (31).

There are two groups of independent continuous random variables *X* = {*x*_1_,*x*_2_,…,*x*_*n*_} and *Y* = {*y*_1_,*y*_2_,…,*y*_*m*_}. The mean of variables are μ_*X*_ and μ_*Y*_. The total size of variables is *m* + *n*. Suppose that *X* and *Y* are identical. The distributions of the two variables have similar shapes. To test whether there is a significant difference between μ_*X*_ and μ_*Y*_, the null hypothesis and the alternative hypothesis can be described as follows:


(1) $$H_{0}:\mu_{X}=\mu_{Y}$$

(2) $$H_{1}:\mu_{X}\neq \mu_{Y}$$

Then, the statistical value of *X* can be calculated as follows:


(3) $$U_{X}\,W_{Y\,X}\,\sum_{i=1}^{m}\left(R_{i-1}-i\right)$$

where *i* represents the rank of rearranged *X* from largest to smallest and *R*_*i–1*_ represents the rank of variable *i-1* in new samples, which mixes *X* and *Y* and rearranged them from largest to smallest. Then, the statistical value of Y can be calculated as follows:


(4) $$U_{Y}=W_{X\,Y}=\sum_{j=1}^{n}\left(R_{j-1}-j\right)$$

The area under the curve (AUC) is used to measure the significance of the difference between two variables. It can be described as follows:


(5) $$\text{AUC}=\frac{U_{X}}{U_{x}+U_{Y}}$$

## Results

### Heat Maps of Visual Attention

**FIGURE 6:**
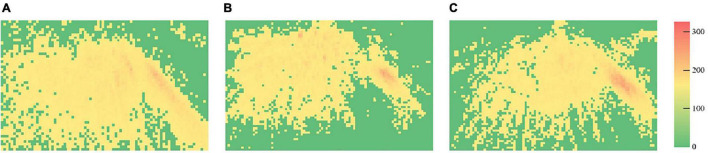
Heat maps of course 1 (**A**, heat map of subject 1; **B**, heat map of subject 2; and **C**, heat map of subject 3).

**FIGURE 7:**
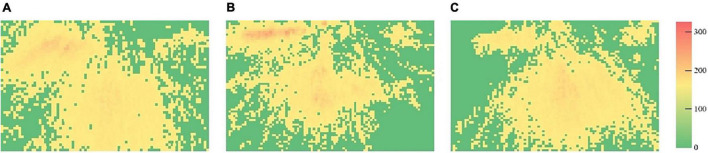
Heat maps of course 2 (**A**, heat map of subject 1; **B**, heat map of subject 2; and **C**, heat map of subject 3).

**FIGURE 8:**
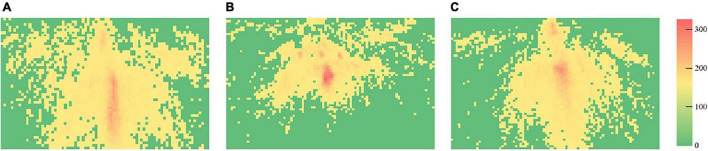
Heat maps of course 3 (**A**, heat map of subject 1; **B**, heat map of subject 2; and **C**, heat map of subject 3).

**FIGURE 9:**
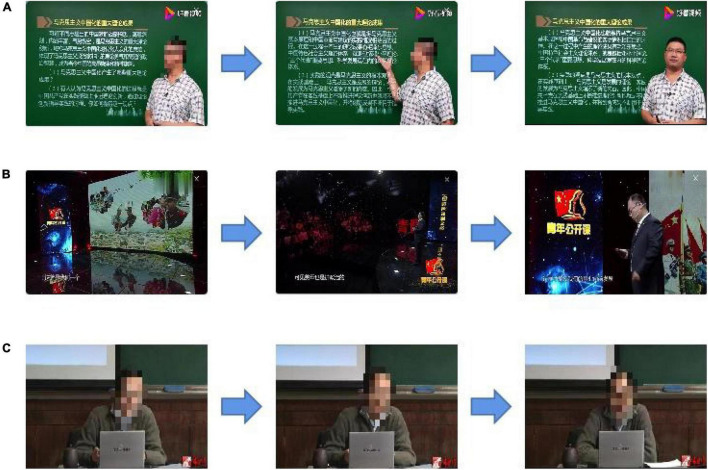
Frame of videos (**A**, course 1; **B**, course 2; and **C**, course 3).

The heat maps of courses are demonstrated in Figures 6–8, respectively, and the frame of the three videos is shown in Figure 9. The maximum value in Figure 6 is 186, and the standard deviation is 12.69. The maximum value in Figure 7 is 303, while the standard deviation is 14.79. The maximum value in Figure 8 is 202, and the standard deviation is 12.53. The minimal value of the three subjects is 0. In the same course, the gaze points have similar distributions for the subjects. They focus on where the text and the teacher appeared in course 1, the gaze points of course 2 are distributed in the teacher and animation areas, and the attention at the subtitle area was less than that of the text area in course 1. The gaze points of the subjects gathered in the teacher’s area. Compared with the previous courses, the heat maps of course 3 were more evenly distributed in the whole screen.

### Ratio of Attention

**FIGURE 10:**
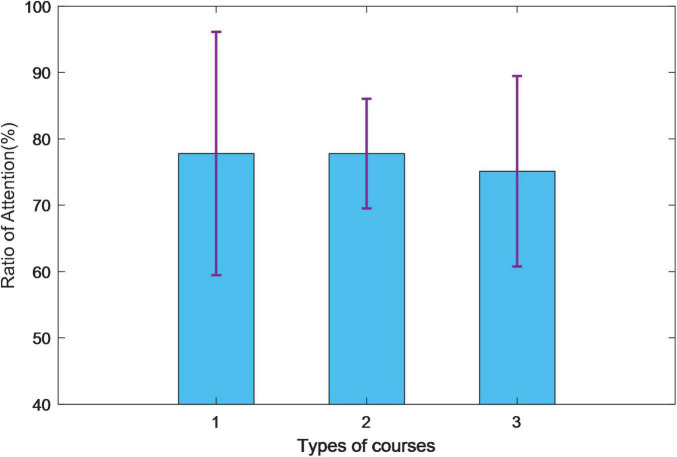
Comparison of attention ratio.

The comparsion of attention are shown in the Table 1 and Figure 10. In course 1, the average time of all subjects’ eyes on the screen is 77.79%. In course 2, the average ratio on the screen is 77.78%. In course 3, it is 75.11%. The variance of the course 1 results was the largest, and the highest attention result and the lowest attention result appear in course 1. The results of course 2 have a small variance, and the distribution is relatively even. The subjects’ attention is lower than in the other two courses.

**TABLE 1:** Comparison of attention ratio.

	Course 1 (%)	Course 2 (%)	Course 3 (%)
Subject 1	62.60	71.75	65.24
Subject 2	72.63	74.36	68.54
Subject 3	98.15	87.23	91.56
Average	77.79	77.78	75.11

**FIGURE 11:**
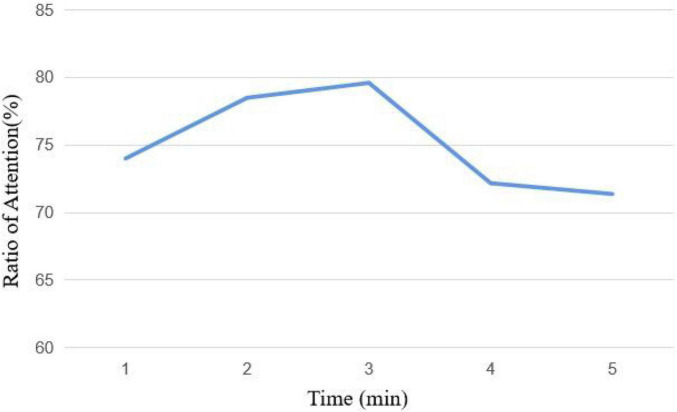
Attention curve of course 1.

The average attention curve in course 1 is shown in Figure 11, which is the same as the change of the human learning attention curve. Attention gradually rises in the first 3 min and then begins to fall in the last 2 min.

**FIGURE 12:**
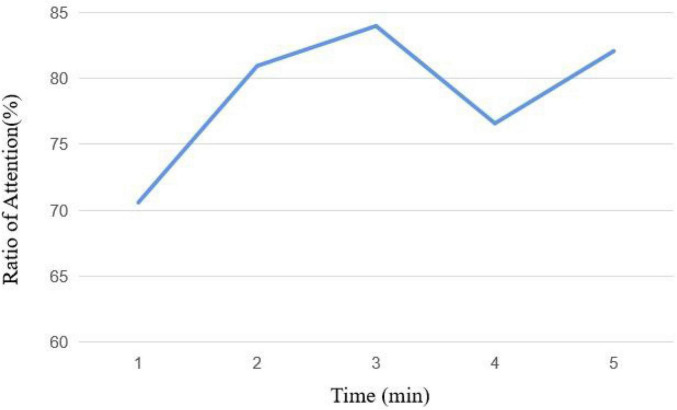
Attention curve of course 2.

The average attention curve in course 2 is shown in Figure 12. The change of the first 4 min in course 2 is the same as the first 3 min in course 1. The attention ratio gradually rises and then falls, but it rises again at the fifth minute. The video uses animation and camera movement technology at the last 2 min to effectively attract the attention of the subjects and increase the attention time on the screen.

**FIGURE 13:**
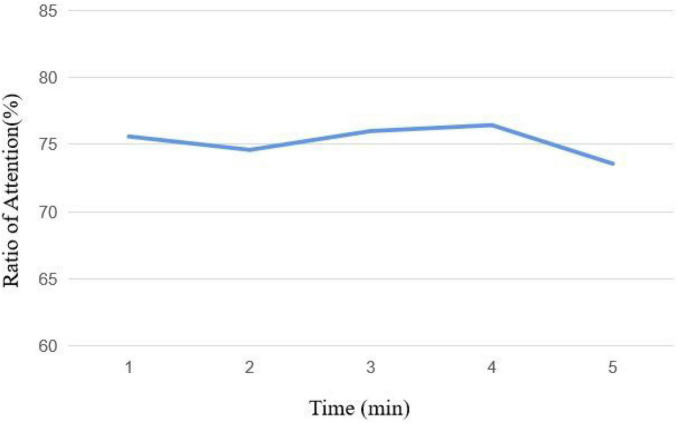
Attention curve of course 3.

The average attention curve in course 3 is shown in Figure 13. In course 3, the attention of the subjects decreased in the second minute and then decreased again after rising. It shows that simple speech without any text can easily make listeners lose interest quickly and reduce their attention. Compared with the previous two courses, the subjects’ attention in course 3 is also the lowest.

### Results of Mann–Whitney Test

**FIGURE 14:**
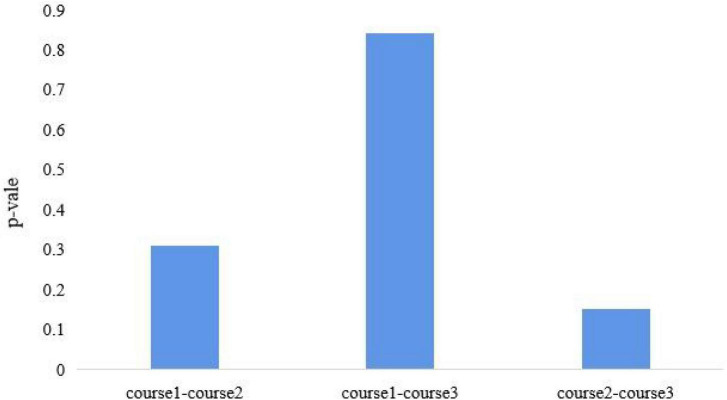
*P*-value of courses.

Mann–Whitney test is used to test two samples from continuous distributions, which have equal medians. A high *P*-value indicates that the two samples have similar distributions. The results are shown in Figure 14. The *P*-value of course 1 and course 2 is 0.3950. The *P*-value of course 1 and course 3 is 0.8413 and that of course 2 and course 3 is 0.1508. It shows that course 1 and course 3 have similar distributions and that course 2 is different from course 1 and course 3. Compared with course 2, the changes of attention of the subjects are more similar. It also shows that animation can improve the attention of the subjects.

## Conclusion

Online lectures become more and more popular during the COVID-19 pandemic period, which prevents the spread of respiratory infection that is likely to happen in the traditional face-to-face teaching mode. To find out the appropriate online teaching format, this study investigated the students’ visual attention during the online lecture process.

If the learning content is presented in the form of written or encoded text, then the information enters from the eyes and is processed in the visual channel, which causes the visual channel to be overloaded, while the other channel does not need to work. The experimental results show that the presentation of words in the form of text and commentary can improve the learning effect on the students.

Animation can attract students to learn and effectively increase their time spent on the screen, but it also affects the time that the subjects spend on watching subtitles. Interspersed and added animation effects during the time when their attention is failing can improve the teaching effect.

## Data Availability Statement

The raw data supporting the conclusions of this article will be made available by the authors, without undue reservation.

## Ethics Statement

Ethical review and approval was not required for the study on human participants in accordance with the local legislation and institutional requirements. Written informed consent for participation was not required for this study in accordance with the national legislation and the institutional requirements. Written informed consent was obtained from the individual(s) for the publication of any potentially identifiable images or data included in this article.

## Author Contributions

QG designed the theoretical framework and collected the data. SL analyzed the data. Both authors wrote the manuscript.

## Conflict of Interest

The authors declare that the research was conducted in the absence of any commercial or financial relationships that could be construed as a potential conflict of interest.

## Publisher’s Note

All claims expressed in this article are solely those of the authors and do not necessarily represent those of their affiliated organizations, or those of the publisher, the editors and the reviewers. Any product that may be evaluated in this article, or claim that may be made by its manufacturer, is not guaranteed or endorsed by the publisher.
